# TWIST-1 promotes cell growth, drug resistance and progenitor clonogenic capacities in myeloid leukemia and is a novel poor prognostic factor in acute myeloid leukemia

**DOI:** 10.18632/oncotarget.4007

**Published:** 2015-05-20

**Authors:** Nan Wang, Dan Guo, YangYang Zhao, ChengYa Dong, XiaoYan Liu, BinXia Yang, ShuWei Wang, Lin Wang, QingGuo Liu, Qian Ren, YongMin Lin, XiaoTong Ma

**Affiliations:** ^1^ State Key Laboratory of Experimental Hematology, Institute of Hematology and Blood Diseases Hospital, Chinese Academy of Medical Sciences, Peking Union Medical College, China

**Keywords:** TWIST-1, myeloid leukemia, leukemia stem cell, prognostic factor, c-MPL

## Abstract

Alterations of TWIST-1 expression are often seen in solid tumors and contribute to tumorigenesis and cancer progression. However, studies concerning its pathogenic role in leukemia are scarce. Our study shows that TWIST-1 is overexpressed in bone marrow mononuclear cells of patients with acute myeloid leukemia (AML) and chronic myeloid leukemia (CML). Gain-of-function and loss-of-function analyses demonstrate that TWIST-1 promotes cell growth, colony formation and drug resistance of AML and CML cell lines. Furthermore, TWIST-1 is aberrantly highly expressed in CD34^+^CD38^−^ leukemia stem cell candidates and its expression declines with differentiation. Down-modulation of TWIST-1 in myeloid leukemia CD34^+^ cells impairs their colony-forming capacity. Mechanistically, c-MPL, which is highly expressed in myeloid leukemia cells and associated with poor prognosis, is identified as a TWIST-1 coexpressed gene in myeloid leukemia patients and partially contributes to TWIST-1-mediated leukemogenic effects. Moreover, patients with higher TWIST-1 expression have shorter overall and event-free survival (OS and EFS) in AML. Multivariate analysis further demonstrates that TWIST-1 overexpression is a novel independent unfavourable predictor for both OS and EFS in AML. These data highlight TWIST-1 as a new candidate gene contributing to leukemogenesis of myeloid leukemia, and propose possible new avenues for improving risk and treatment stratification in AML.

## INTRODUCTION

Acute myeloid leukemia (AML) is a heterogeneous clonal malignancy with considerable diversity concerning clinical behavior and prognosis [1–3]. Chronic myeloid leukemia (CML) is a hematopoietic stem cell disease characterized by constitutive activation of the BCR-ABL tyrosine kinase [4]. Despite this consistent molecular abnormality, CML still exhibits marked heterogeneity in prognosis because of development of secondary cytogenetic changes or resistance [5, 6]. In recent years, although many of genetic and molecular alterations, such as deregulation of gene expression or acquired genetic mutation, have proven to be useful molecular tools for diagnosis and risk stratification of myeloid leukemia, the discovery and validation of new discriminative biomarkers remain of utmost value to improve outcome prediction or provide potential targets for therapy.

TWIST-1, a highly conserved basic helix-loop-helix transcription factor, plays a key role in the specification and differentiation of the tissues with a mesodermal origin in both *Drosophila* and vertebrates [7–9]. In human, overexpression of TWIST-1 has been observed in various solid tumors and is often associated with aggressive phenotypes and poor prognosis [10–14]. It's now well accepted that TWIST-1, which may function as a multifunctional proto-oncogene during tumorigenesis and progression of solid tumors, protects cells from chemotherapy-induced apoptosis and senescence and promotes tumor epithelial-mesenchymal transition [13, 15–19].

In many cancers, evidence suggests that a small subset of malignant cells, termed cancer stem cells (CSCs), is wholly responsible for tumor propagation, metastasis, disease relapse and drug resistance. Targeting of CSCs carries the hope of curing cancer [20]. Recently, TWIST-1 has attracted intense interest due to its contribution in generation and maintenance of CSCs. Overexpression of TWIST-1 in breast cell lines, head and neck squamous cell carcinoma cells, and cervical cancer cells enhanced tumor-initiating and self-renewal capability [21–23]. In the blood system, our previous study demonstrates that TWIST-1 is highly expressed in mouse long-term hematopoietic stem cells (LT-HSCs) and is a novel regulator of HSC self-renewal and myeloid lineage development [24]. Thus far, there are only a few studies concerning the role of TWIST-1 in human hematopoietic malignancies. Cosset et al reveals that overexpression of TWIST-1 represents a prognostic factor in CML and may contribute to drug resistance [25]. TWIST-1 has also been reported as an antiapoptotic factor in myelodysplastic syndromes (MDS) [26]. However, the role of TWIST-1 in AML and acute lymphoid leukemia (ALL), whether it is associated with leukemia stem cells (LSCs), and its potential pathogenic mechanism in CML remain unknown.

We first determined TWIST-1 expression level by quantitative real-time PCR and immunohistochemical (IHC) in different hematopoietic malignancies including AML, ALL and CML. Our study demonstrated that TWIST-1 was highly expressed in bone marrow mononuclear cells (BMMNCs) of patients with AML and CML, whereas normalization of TWIST-1 expression was observed in patients with ALL. We also found that TWIST-1 enhanced cell growth, colony formation, drug resistance and tumor formation in AML and CML cell lines. In addition, we analyzed TWIST-1 expression patterns in different hematopoietic cell populations from AML and CML patients, and found that TWIST-1 was most highly expressed in CD34^+^CD38^−^ cells but showed a low abundance in more differentiated descendants. TWIST-1 knockdown impaired stem/progenitor cell colony-forming capacity of primary myeloid leukemia CD34^+^ cells. Furthermore, TWIST-1 could mediate the expression of c-MPL by interfering with RUNX1. Overexpression of c-MPL could significantly attenuate the inhibitory effects of knockdown TWIST-1 on the growth of AML and CML cell lines. TWIST-1 overexpression resulted in the activation of phosphorylation of the PI3K/AKT and JAK2/ERK pathways which are downstream pathways of c-MPL. These results suggested a functional interaction between TWIST-1 and c-MPL in AML and CML cell lines. Most importantly, we identified TWIST-1 as a novel independent prognostic factor for poor outcome in AML.

## RESULTS

### Overexpression of TWIST-1 in myeloid leukemia cell lines and patients with AML and CML

**Figure 1:**
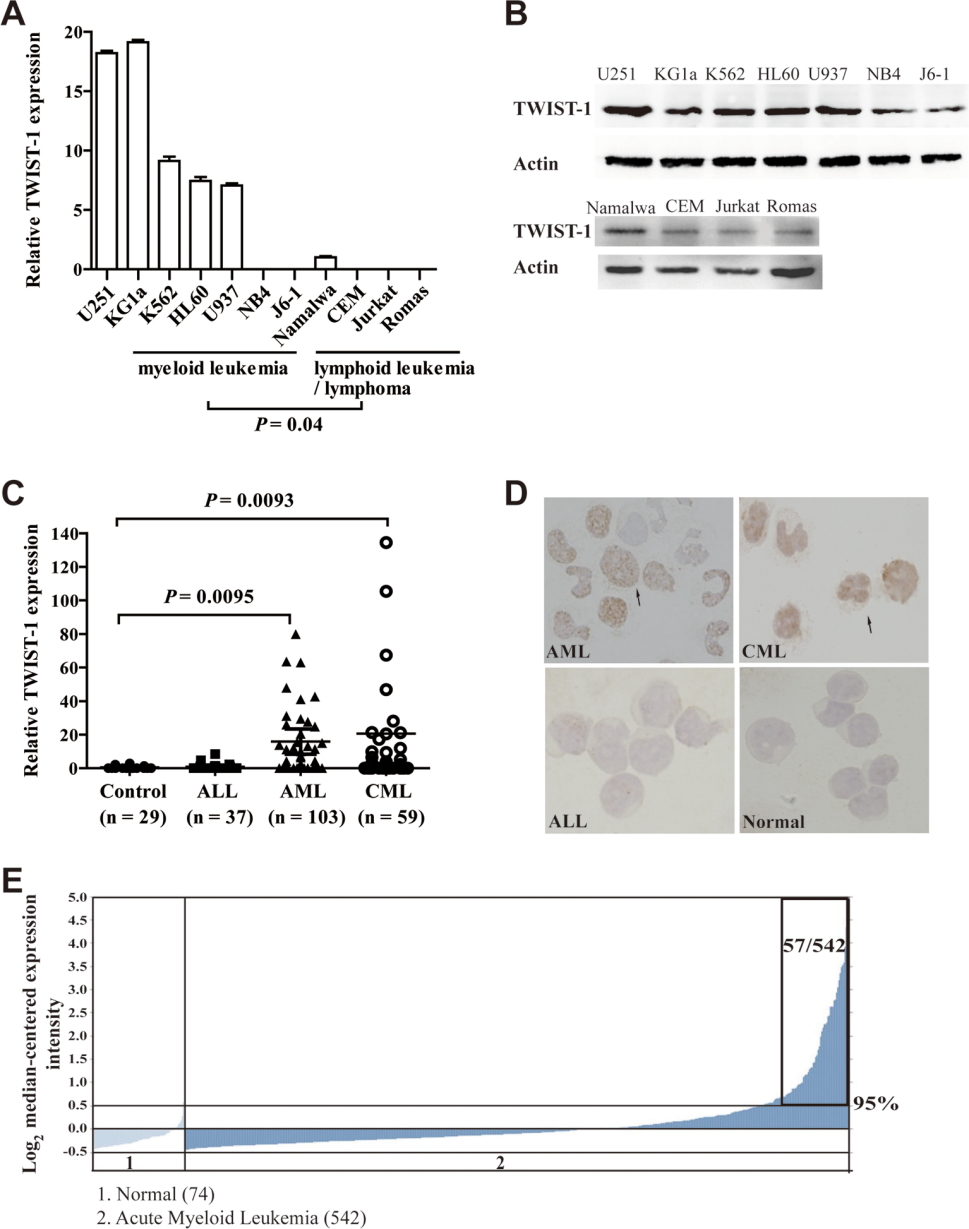
TWIST-1 is overexpressed in myeloid leukemia cell lines and patients with AML and CML **A.** Relative expression of TWIST-1 mRNA in hematopoietic malignant cell lines using quantitative real-time PCR. Data are shown as the mean ± SEM of three independent experiments, endogenous reference was GAPDH. **B.** Western blot analysis of TWIST-1 protein levels in hematopoietic malignant cell lines. β-Actin was shown as equal loading. **C.** TWIST-1 mRNA expression in BMMNCs from patients with ALL, AML and CML, as determined by quantitative PCR. BMMNCs from healthy volunteers and patients with idiopathic thrombocytopenic purpura (ITP) were used as controls. GAPDH served as an internal control. Horizontal bars denote the medians. **D.** Immunohistochemical staining of BMMNCs at presentation from patients with AML, CML and ALL, and a healthy volunteer for TWIST-1. Specimens from AML and CML patients with higher TWIST-1 expression showed strong staining and from an ALL patient and a healthy volunteer with lower TWIST-1 expression showed weak staining. Magnification × 100, respectively. **E.** Caner Outlier Profile Analysis revealed TWIST-1 as a gene with outlier expression profile at the 95th percentile in Haferlach et al.'s AML dataset. TWIST-1 expression is shown from all profiled samples in this dataset. The microarray data indicate that TWIST-1 is highly overexpressed in a subset of AML samples (57/542). The boxed region indicates 57 AML samples with overexpression of TWIST-1 among 542 patients examined. “Normal” group includes healthy volunteer samples. Visualization tools incorporated in Oncomine were used to generate graphical displays.

To determine the potential role of TWIST-1 in leukemia, we quantified the mRNA and protein expression of TWIST-1 in the myeloid cell lines NB4, KG1a, J6–1, U937, HL-60, and K562, originally derived from patients with myeloid leukemia, as well as CEM, Ramos, Jurkat, and Namalwa derived from leukemia of lymphoid origin, or lymphoma patients. The human glioma cell line U251 was used as a positive control for TWIST-1 detection [27]. We observed significantly higher TWIST-1 in myeloid compared with lymphoid cell lines (Figure 1A–1B). Next, we analyzed primary leukemia samples and collected BMMNCs derived from patients with AML (*n* = 103), CML (*n* = 59) and ALL (*n* = 37). In spite of the wide range of individual values of TWIST-1, median levels of TWIST-1 were significantly higher in patients with AML and CML than in controls (*n* = 29), whereas no significant difference was observed in TWIST-1 expression between ALL patients and controls (Figure 1C). Furthermore, TWIST-1 protein expression measured by scoring IHC of BM samples correlated well with TWIST-1 mRNA expression in the 31 patients studied (Supplementary Figure S1), suggesting that TWIST-1 mRNA expression was informative in the prediction of protein expression. Representative of IHC of AML and CML BMMNCs with higher scores and ALL and healthy volunteer BMMNCs with lower scores were shown in Figure 1D. In order to further substantiate our findings that TWIST-1 was overexpressed in myeloid leukemia, we analyzed TWIST-1 expression using several published datasets from the Oncomine databases. We found that TWIST-1 transcript levels are significantly upregulated in AML samples based on microarray gene expression data of human AML (*n* = 542) and healthy individuals (*n* = 74) (Figure 1E) [28, 29].

We then analyzed the correlation of TWIST-1 expression with other clinical characteristics of leukemia. All patient data were shown in Supplementary Table S2. We found that the expression level of TWIST-1 was not related to sex, age, white blood cell counts, BM blasts, extramedullary infiltration or CD34 expression in AML and CML samples (Supplementary Tables S3 and S4). Of note, patients with high TWIST-1 expression had a higher percentage of FAB M3 subtype in AML (Supplementary Table S3).

Overall, the results reveal that aberrant high expression level of TWIST-1 is found in patients with AML and CML, suggesting a possible role of TWIST-1 in myeloid leukemogenesis.

### Enforced TWIST-1 expression leads to enhanced cell growth and drug resistance

**Figure 2:**
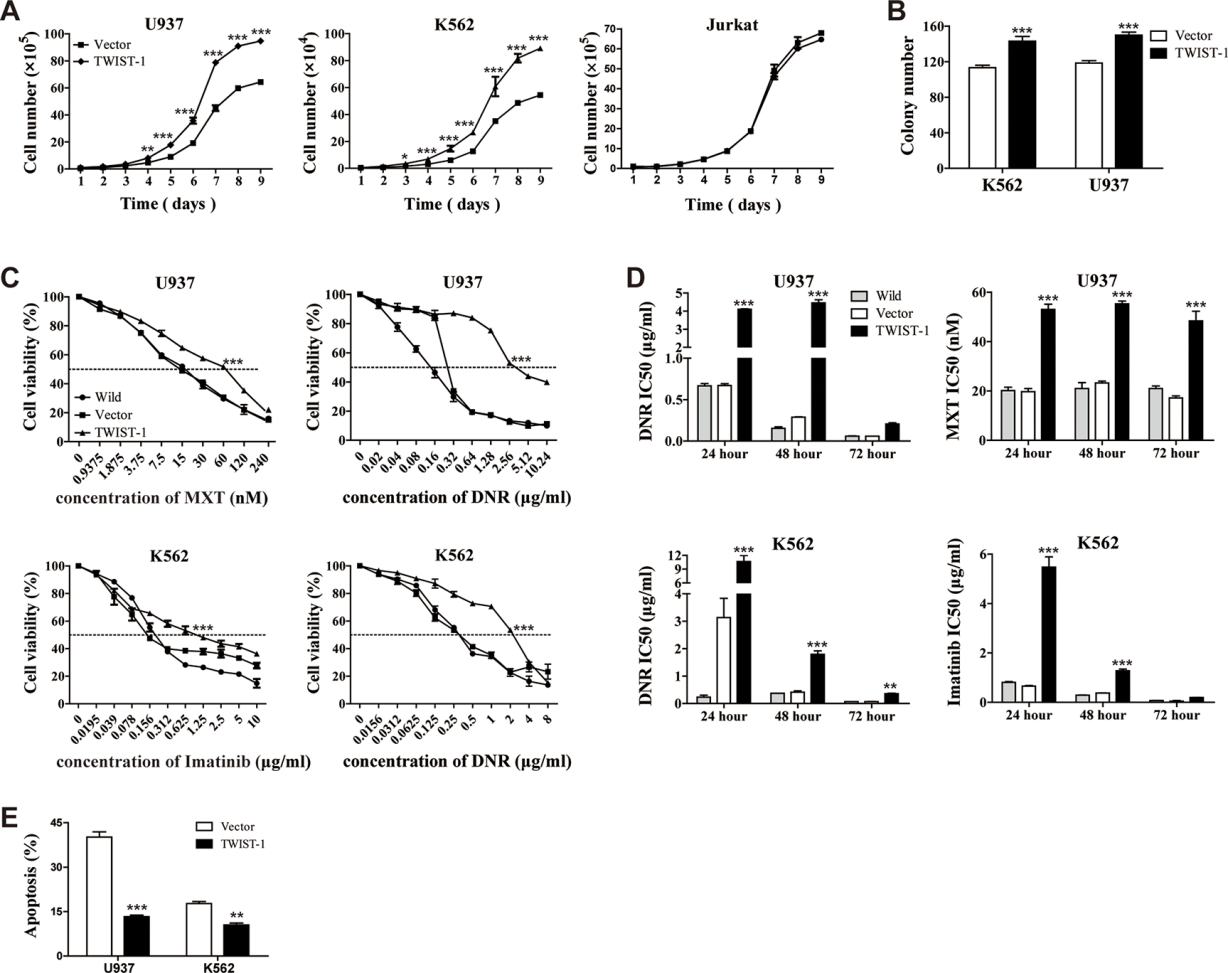
Overexpression of TWIST-1 enhances cell proliferation, colony formation and drug resistance in K562 and U937 cells U937 and K562 cells transfected with TWIST-1 or vector were used in the following experiments. **A.** Cell proliferation was generated by seeding and counting transduced cells of U937, K562 and Jurkat in triplicate using a hemocytometer under light microscopy by trypan blue exclusion method. **B.** Representative quantitation from colony formation assay. **C.** Transduced U937 and K562 cell lines were treated with various concentrations of DNR and MXT, or DNR and Imatinib respectively for 48 hours. Cell viability was measured by MTT assay and expressed as a percentage relative to control cells. IC50 values were calculated with XLfit software, which are marked in the middle of the box. **D.** IC50 values of DNR and MXT in U937 cells and DNR and Imatinib in K562 cells are illustrated in bar graph. **E.** Transfected U937 and K562 cells were treated with 0.78 μg/ml DNR and 1 μg/ml Imatinib, respectively. Apoptosis was analyzed by Annexin V staining. The bar graph represents the percentage of apoptotic cells. Data are presented as mean ± SEM of three independent experiments. Asterisks denote significance (*, *P* < 0.05; **, *P* < 0.01; ***, *P* < 0.001).

The above results prompted us to investigate the role of TWIST-1 in tumorigenesis. We used a pCDH1-based lentiviral system to mediate transduction of TWIST-1 cDNA-containing expression vector or empty vector into U937 and K562 cells. Cell lines that stably expressed TWIST-1 were generated and analyzed by real-time PCR and Western blot analysis (Supplementary Figure S2A–S2B). Overexpression of TWIST-1 significantly promoted cell growth and colony formation in both U937 and K562 cells, while no difference in cell growth was detected in Jurkat cells (Figure 2A–2B). In recent years, emerging evidences suggest that TWIST-1 enhances the acquired resistance of cancer cells to drug [16, 18, 30, 31]. To further evaluate the biological significance of TWIST-1 on the cell sensitivity to drug in myeloid leukemia cells, a cell viability assay was then performed. Compared with U937 cells and vector alone-transduced U937 cells, TWIST-1-transduced U937 cells showed a significant decrease in cell sensitivity to Daunorubicin (DNR) and Mitoxantrone (MXT) over 24, 48 and 72 hours. TWIST-1-transducd U937 cells were 4- to 17-fold more resistant to DNR and 2- to 3-fold more resistant to MXT than cells expressing control vector (Figure 2C–2D and Supplementary Figure S2C). Similar phenomenon was observed in K562 cells. When treated with Imatinib and DNR, TWIST-1-transducd K562 cells significantly increased resistance at different time points (Figure 2C–2D and Supplementary Figure S2C).

Meanwhile, we examined the protection activity of TWIST-1 on apoptosis. Transfected U937 and K562 cells were treated with DNR and Imatinib, respectively, for 24 hours to induce apoptosis. We found that TWIST-1 significantly decreased the apoptotic rate of U937 and K562 cells (Figure 2E). Although overexpression of TWIST-1 did not directly affect apoptosis of U937 and K562 (data not shown), it provided protection activity against apoptosis induced by DNR and Imatinib. In addition, TWIST-1 induced the accumulation of U937 cells in the S phase of the cell cycles, but not in K562 cells (Supplementary Figure S2D). These results strongly suggest that TWIST-1 promotes cell growth, colony formation and drug resistance in myeloid leukemia.

### Knockdown of TWIST-1 inhibits tumor growth

**Figure 3:**
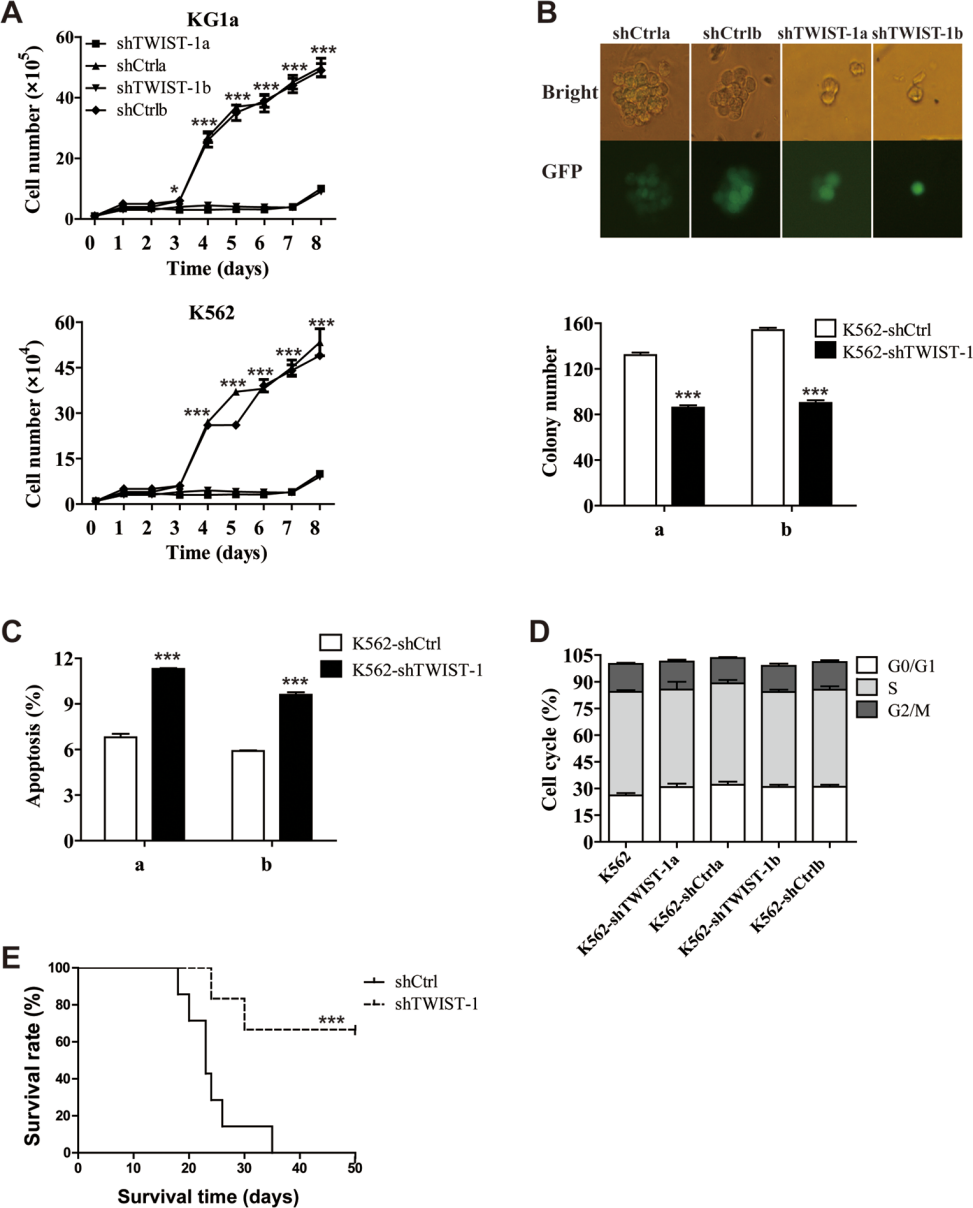
shRNA-mediated silencing of TWIST-1 expression inhibits the tumor growth **A.** Cell proliferation of TWIST-1-silenced cells or shCtrl control cells was monitored at different time points. **B.** Morphology of colonies and colony numbers derived from K562 cells transduced with shTWIST-1a or shTWIST-1b compared with the controls. **C.** TWIST-1 knockdown-induced apoptosis was assessed in K562 cells using an Annexin V/7AAD staining. The bar graph represents the percentage of apoptotic cells. **D.** Cell-cycle distribution of TWIST-1-silenced cells and non-silencing control cells was analyzed by propidium iodide staining. **E.** The Kaplan-Meier survival curve of NOD/SCID mice. Data are presented as mean ± SEM. Asterisks denote significance (*, *P* < 0.05; **, *P* < 0.01; ***, *P* < 0.001).

In order to further identify the potential function of TWIST-1, we silenced TWIST-1 in K562 and KG1a cell lines. Robust TWIST-1 knockdown was achieved following lentiviral transduction with pLL3.7-based vector expressing either shTWIST-1 or scrambled sequence only as a control. Knockdown of TWIST-1 was confirmed by real-time PCR and Western blotting analysis in transduced K562 cells (Supplementary Figure S3A–S3B). Silencing of TWIST-1 decreased cell growth over time in both KG1a and K562 cell lines (Figure 3A). The reduction of TWIST-1 expression also resulted in a significant decrease in the number and size of colonies in K562 cells (Figure 3B). In addition, flow cytometry analysis showed increased apoptosis in shTWIST-1-transduced K562 cells (Figure 3C), suggesting the important role of TWIST-1 in the survival of leukemia cells. Although no differences in cell cycle were detected in K562 cells (Figure 3D), TWIST-1 ablation induced cell cycle arrest at G1 phase in U937 cells (Supplementary Figure S3C), suggesting that TWIST-1 impacts the cell cycle distribution in a cell type dependent manner.

To confirm the *in vivo* tumorigenicity-ability of TWIST-1, K562 cells transfected with shTWIST-1 or shCtrl were injected by tail vein in NOD/SCID mice. The expression of human CD13 and CD33-positive tumor cells, which are considered to be the tumor marker of K562 cells, was high in BM and spleen in dying mice, which suggested that the mouse model bearing leukemia could be established (Supplementary Figure S4A–S4B). We found that the survival time of shTWIST-1 group was significantly longer than that of shCtrl group (Figure 3E). The results indicate that down-regulation of TWIST-1 reduces tumor growth *in vitro* and *in vivo* and this effect is due at least in part to increased cell apoptosis.

### TWIST-1 is predominantly expressed in myeloid leukemia stem cell candidates and TWIST-1 inhibition reduces leukemia stem/progenitor cell frequencies

**Figure 4:**
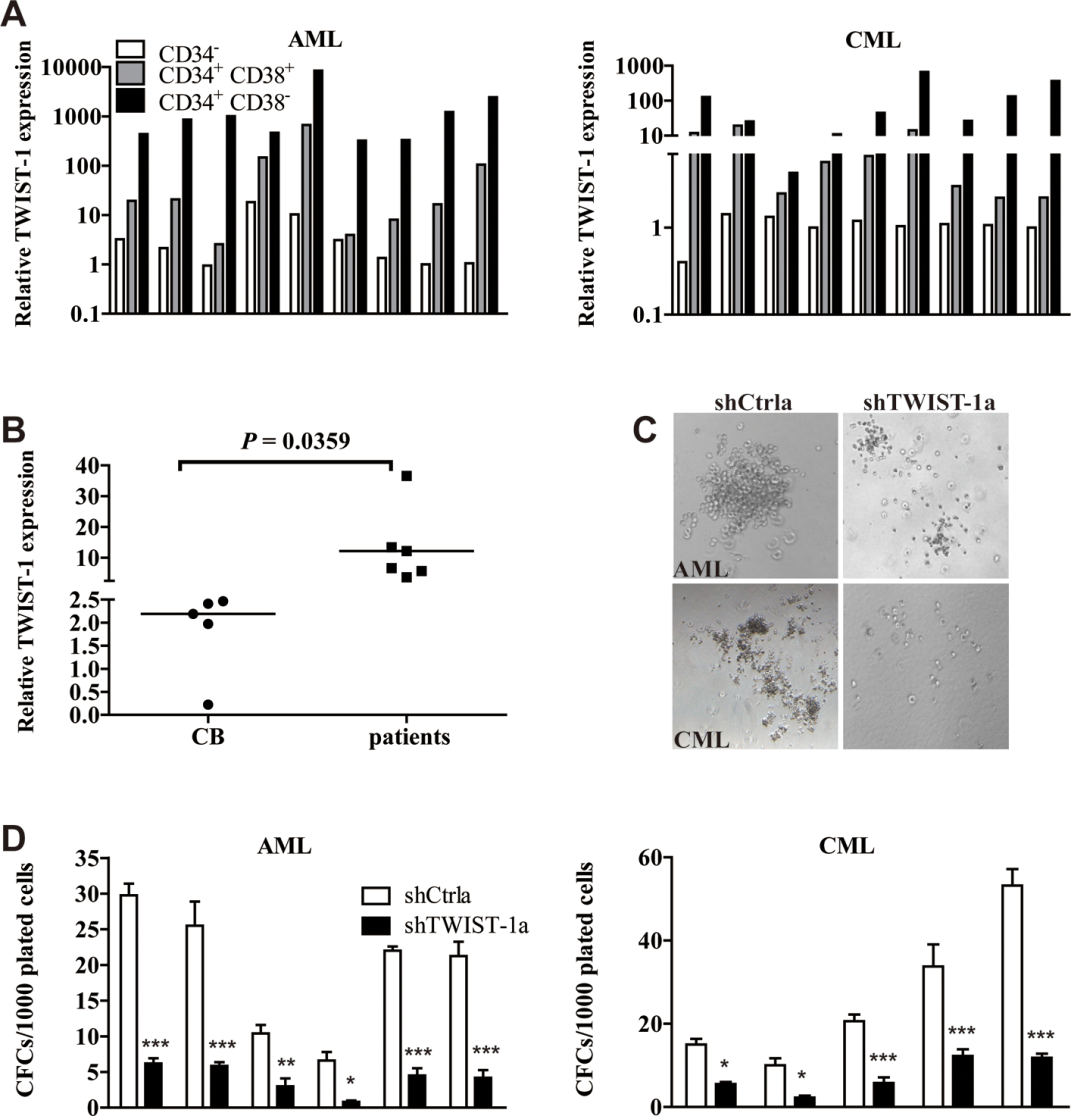
TWIST-1 is highly expressed in myeloid leukemia stem cells and knockdown of TWIST-1 reduces CFC formation of CD34^+^ cells **A.** Expression of TWIST-1 in CD34^+^CD38^−^, CD34^+^CD38^+^ and CD34^−^ subpopulations of patients with AML and CML. Expression analyses were performed by quantitative real-time PCR, and normalized to GAPDH internal control. **B.** Analysis of TWIST-1 expression in CD34^+^CD38^−^ cells from AML and CML patients compared with human CB CD34^+^CD38^−^ cells by quantitative real-time PCR. Endogenous reference was GAPDH. Horizontal bars denote the medians. **C.** CD34^+^ cells from patients with AML and CML were transduced with shCtrl or shTWIST-1 particles, sorted and plated into methylcellulose medium H4434. Representative micrographs of colonies in methylcellulose displayed a reduction in colony size after TWIST-1 knockdown. **D.** After 14 days of culture, colonies were counted. Data are presented as mean ± SEM. Asterisks denote significance (*, *P* < 0.05; **, *P* < 0.01; ***, *P* < 0.001).

Our previous study has shown that TWIST-1 is selectively expressed in mouse LT-HSCs [24]. Cosset [25] and Li [26] have reported that TWIST-1 is highly expressed in the CD34^+^ compartments in patients with CML and MDS. We were, therefore interested in determining the expression of TWIST-1 in immature myeloid leukemia cells. Aberrantly high expression of TWIST-1 in leukemia stem/progenitor cells could be confirmed when we analyzed the expression of TWIST-1 in highly purified leukemic CD34^+^CD38^−^, CD34^+^CD38^+^ and CD34^−^ cells isolated from patients with AML (*n* = 9) and CML (*n* = 9). TWIST-1 was expressed at the highest level in CD34^+^CD38^−^ cells, and its expression levels in both CD34^+^CD38^−^ and CD34^+^CD38^+^ subpopulations were higher than that in CD34^−^ cells (Figure 4A). Importantly, TWIST-1 expression in CD34^+^CD38^−^ immature cells from myeloid leukemia patients (*n* = 6) was significantly higher compared with cord blood (CB) (*n* = 5) (Figure 4B). The findings that TWIST-1 was predominantly expressed in the leukemia stem cell candidates of AML and CML suggest a possible role of TWIST-1 in primitive myeloid leukemia cells.

Therefore, we next investigated the effects of down-modulation of TWIST-1 on the colony forming ability in myeloid leukemia stem/progenitor cells. CD34^+^ cells were isolated from AML (*n* = 6) and CML (*n* = 5) patients, followed by transduction with shCtrl or shTWIST-1 vectors and sorting for green fluorescent protein (GFP) expression. Transduction efficiencies ranged from 10% to 50% for both groups (data not shown). All AML and CML samples studied in CFC were known to harbor the FLT3/ITD and BCR/ABL, respectively. The results showed that not only was the number of colony-forming cells (CFCs) strongly reduced, the size of colonies also was reduced upon TWIST-1 down-modulation (Figure 4C–4D). No significant effects were observed on hematopoietic differentiation upon down-modulation of TWIST-1 (data not shown). We further demonstrated that most of the colonies from the CFC were of AML or CML origin, as determined by FLT3/ITD or BCR/ABL PCR (Supplementary Table S5).

These observations indicate that TWIST-1 is not only highly expressed in myeloid leukemia stem cell candidates, but also is critical for maintenance of leukemia stem/progenitor cell frequencies.

### TWIST-1 regulates c-MPL expression by interfering with RUNX1

**Figure 5:**
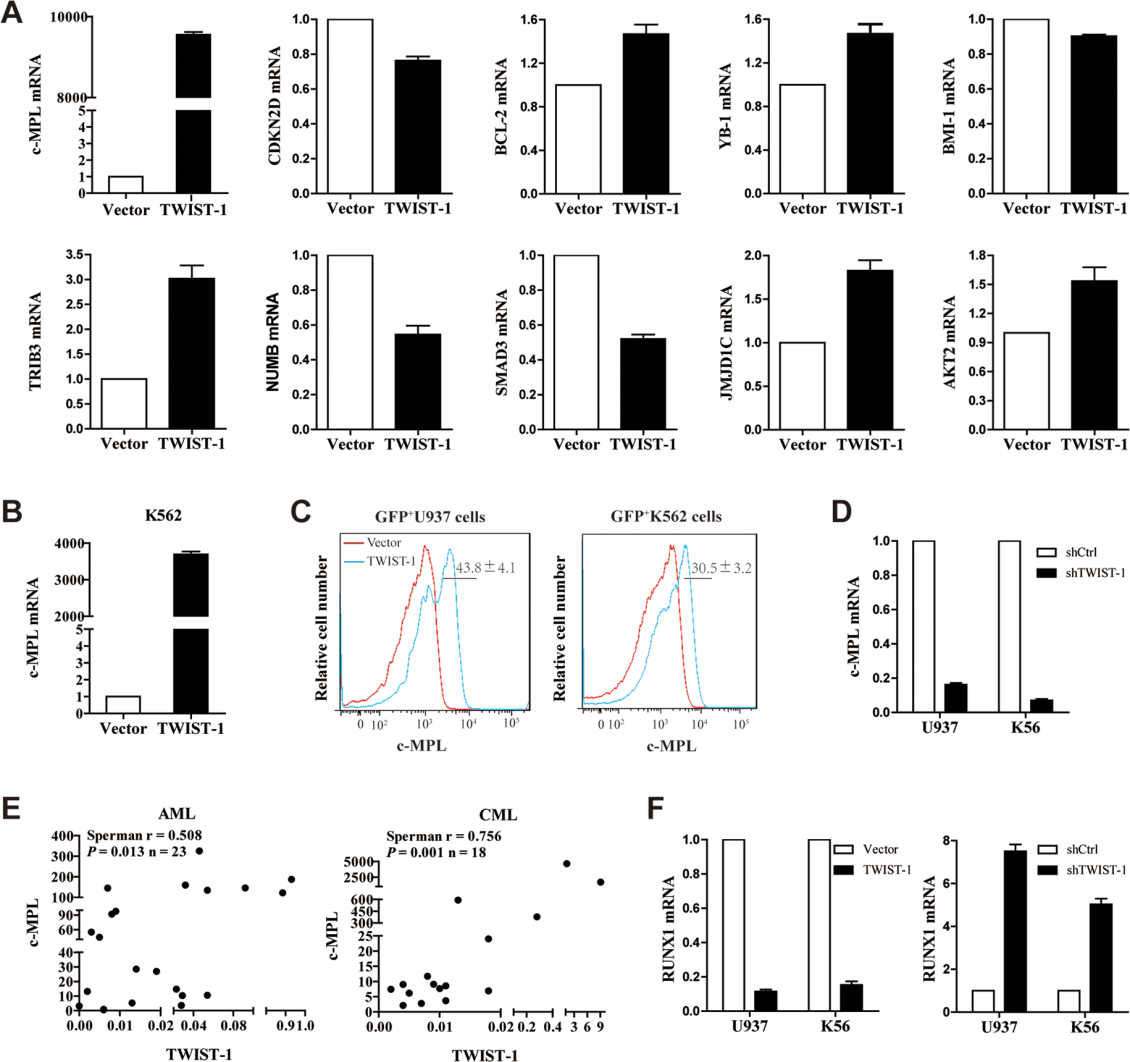
Positive correlation between TWIST-1 and c-MPL expression **A.** U937 cell line was transduced with TWIST-1 or empty vector, and 72 hours after transduction, GFP^+^ cells were sorted and gene expression was analyzed by quantitative real-time PCR. Data are presented as mean ± SEM of three independent experiments. **B.** K562 cell line was transduced with TWIST-1 or empty vector, the expression of c-MPL was analyzed by quantitative real-time PCR. Data are presented as mean ± SEM of three independent experiments. **C.** Analysis of protein expression of c-MPL by FACS. **D.** The expression of c-MPL in TWIST-1 shRNA-transduced K562 and U937 cells was examined by quantitative real-time PCR. Endogenous reference was GAPDH. Data are presented as mean ± SEM of three independent experiments. **E.** The scatter plots represent the correlation of the TWIST-1 and c-MPL mRNA levels in AML and CML samples. **F.** Analysis of RUNX1 mRNA levels by quantitative real-time PCR. Data are presented as mean ± SEM of three independent experiments.

Given the strong effect of TWIST-1 on leukemogenesis, we sought to explore downstream target genes of TWIST-1 in AML and CML. Affymetrix-based gene expression profilings of vector- or TWIST-1-transduced U937 cells uncovered prominent transcriptional changes in the expression of 923 genes, of which 376 were up-regulated and 547 were down-regulated following TWIST-1 overexpression (≥ 1.2-fold deregulation, *P* < 0.05). We selected the most significant biological processes and molecular function altered, which genes were primarily clustered in regulating the processes of the cell growth, drug resistance and leukemia stem cell. Real-time PCR analysis was carried out to validate the array data. Our results showed that the c-MPL mRNA level of TWIST-1-transfected U937 cells became the most significantly increased (Figure 5A). Moreover, the real-time PCR results demonstrated that TRIB3 and JMJD1C mRNA levels were increased approximately three-fold and two-fold, respectively (Figure 5A). The expression of NUMB and SMAD3 were decreased approximately doubled following TWIST-1 overexpression (Figure 5A). c-MPL, a receptor for thrombopoietin, which is not only expressed in different maturation stages of megakaryocte, or early hematopoietic stem/progenitor cells, but also expressed in the primitive cells of AML, CML and MDS patients, promotes the cell proliferation and resistance to chemotherapy of various types of AML cases [32–35]. Our results showed that the c-MPL mRNA level of TWIST-1-transfected K562 cells were also significantly increased (Figure 5B). Analysis of sorted GFP^+^K562 and GFP^+^U937 cells transduced with TWIST-1-containing vectors displayed that the protein levels of c-MPL were obviously up-regulated when compared with controls (Figure 5C). Moreover, TWIST-1 shRNA-transduced U937 and K562 cells showed significant reduction in the expression of c-MPL (Figure 5D). Next, we investigated the correlation between TWIST-1 and c-MPL in BMMNCs from AML (*n* = 23) and CML (*n* = 18) by real-time PCR. Strikingly, the expression of TWIST-1 and c-MPL showed a significant trend toward positive correlation in myeloid leukemia samples (Figure 5E).

By searching the JASPAR database, we found consensus E-box sequence motifs in the promoter region of c-MPL, representing possible binding sites of TWIST-1. To test whether c-MPL can be directly targeted by TWIST-1, we constructed the luciferase reporters that contained the putative TWIST-1 binding sites (Supplementary Figure S5A). Our results showed that compared to negative control, overexpression of TWIST-1 had no effect on the firefly luciferase activity of c-MPL construct (Supplementary Figure S5B), which suggests that c-MPL is not a direct target of TWIST-1. It has been reported that TWIST-1 could directly bind to the RUNX1 Runt domain and decrease RUNX1 transactivation activity [36]. In HSPCs, RUNX1 works as a negative regulator of the c-MPL [37]. We have previously characterized that RUNX1 transcript level is repressed and c-MPL expression is up-regulated in TWIST-1-overexpressing Lin^−^c-Kit^+^Sca-1^+^ (LKS) cells in the mouse hematopoietic system [24]. Thus, we analyzed whether TWIST-1 mediated the expression of c-MPL by RUNX1 in AML and CML cell lines. As expected, a significant repression of RUNX1 transcript level in U937 and K562 overexpressing TWIST-1 was observed compared with the control cells (Figure 5F). Moreover, the expression of RUNX1 in TWIST-1 shRNA-transduced K562 and U937 cells were obviously up-regulated (Figure 5F). Collectively, these studies indicate that the important role for TWIST-1 in AML and CML pathology may involve the RUNX1/c-MPL regulatory pathway.

### TWIST-1 promotes cell growth and colony formation depending on c-MPL

**Figure 6:**
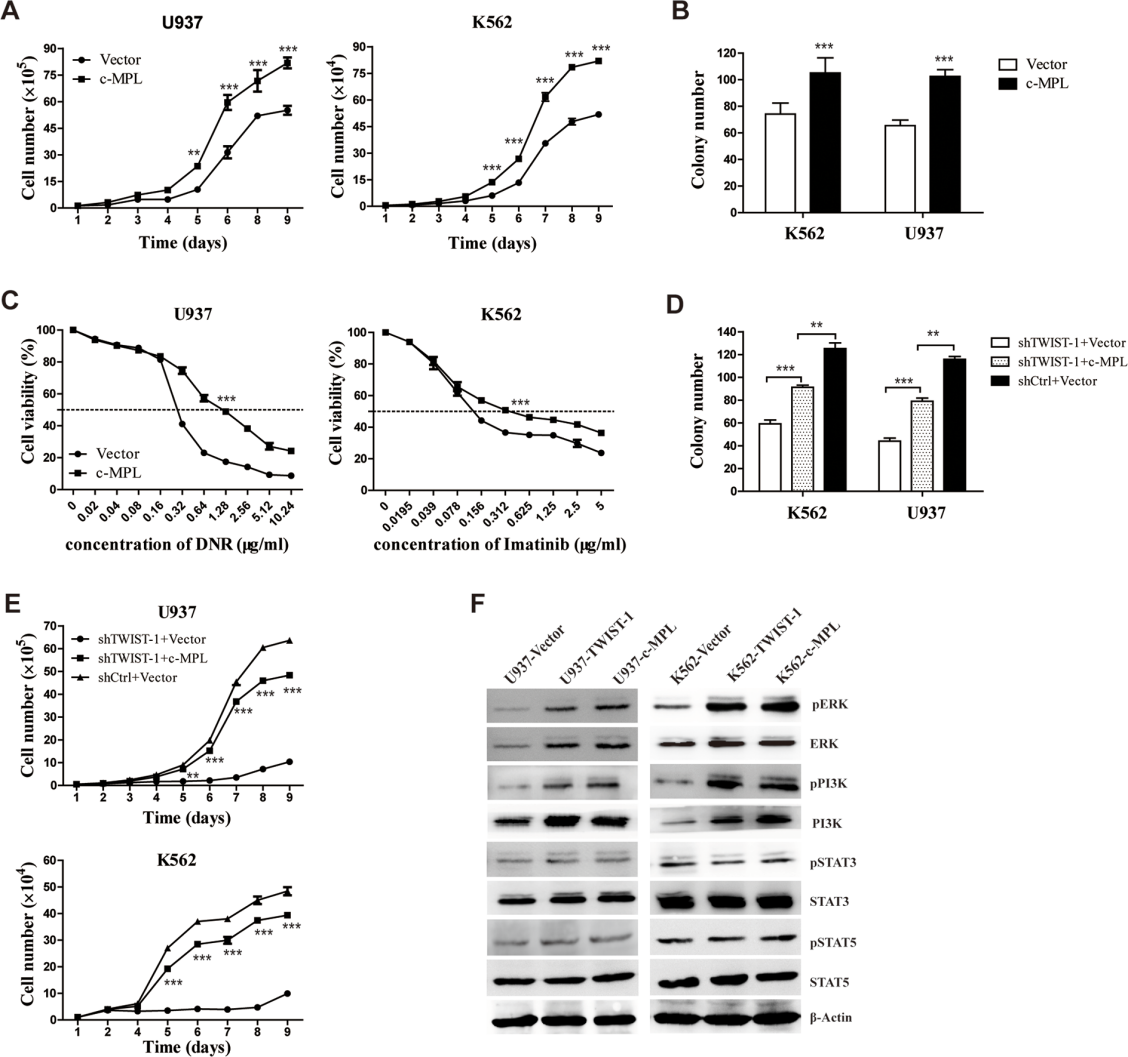
TWIST-1 promotes cell growth and drug resistance depending on c-MPL **A–C.** Cell proliferation (A), colony formation (B) and drug resistance (C) of U937 and K562 cells transfected with c-MPL or empty vector were determined by cell counting, colony-forming assay and cell viability assay, respectively. **D–E.** Control or shTWIST-1 transfected U937 and K562 cells were further transduced with retrovirus vectors expressing c-MPL-BFP or vector-BFP. Colony formation (D) and cell proliferation (E) were determined by colony-forming assay and cell counting, respectively. **F.** Western blot analysis of phospho-ERK, phospho-PI3K, phospho-STAT3, phospho-STAT5, ERK, PI3K, STAT3 and STAT5 in the TWIST-1 and c-MPL-transduced K562 and U937 cells. Phosphorylation protein was examined by fold change over total protein and normalized to β-Actin. Asterisks denote significance (**, *P* < 0.01; ***, *P* < 0.001).

To determine the functional interaction of TWIST-1 and c-MPL in regulating myeloid leukemia, we first examined the effect of c-MPL overexpression in U937 and K562 cells. Cell lines that stably expressed c-MPL were generated and analyzed by FACS (Supplementary Figure S6). Consistent with the results from TWIST-1 overexpression studies, the cell growth and CFC production of U937 and K562 cells were promoted when c-MPL was overexpressed (Figure 6A–6B). Moreover, c-MPL-transduced U937 and K562 cells also showed a significant decrease in cell sensitivity to DNR and Imatinib (Figure 6C). Next, we investigated whether exogenous expression of c-MPL using a c-MPL expression plasmid rescued the impaired cell growth and colony formation caused by TWIST-1 knockdown. Our results showed that enforced expression of c-MPL partially recovered the cell growth and colony formation ability in TWIST-1 knockdown cells (Figure 6D–6E).

We also determined whether the downstream signaling pathways of c-MPL underlie the TWIST-1-mediated effects in myeloid leukemia. We demonstrated that TWIST-1 overexpression in U937 and K562 cell lines led to 1.4-fold and 1.6-fold higher contents of ERK phosphorylation, and 2-fold and 1.8-fold higher contents of PI3K phosphorylation, respectively. However, TWIST-1 overexpression did not induce STAT3 and STAT5 activation. These observations are consistent with the effects of c-MPL overexpression (Figure 6F). These results indicate that TWIST-1-mediated leukemogenesis is partially due to regulation of c-MPL expression.

### Higher TWIST-1 expression is an independent poor prognostic factor in AML

**Figure 7:**
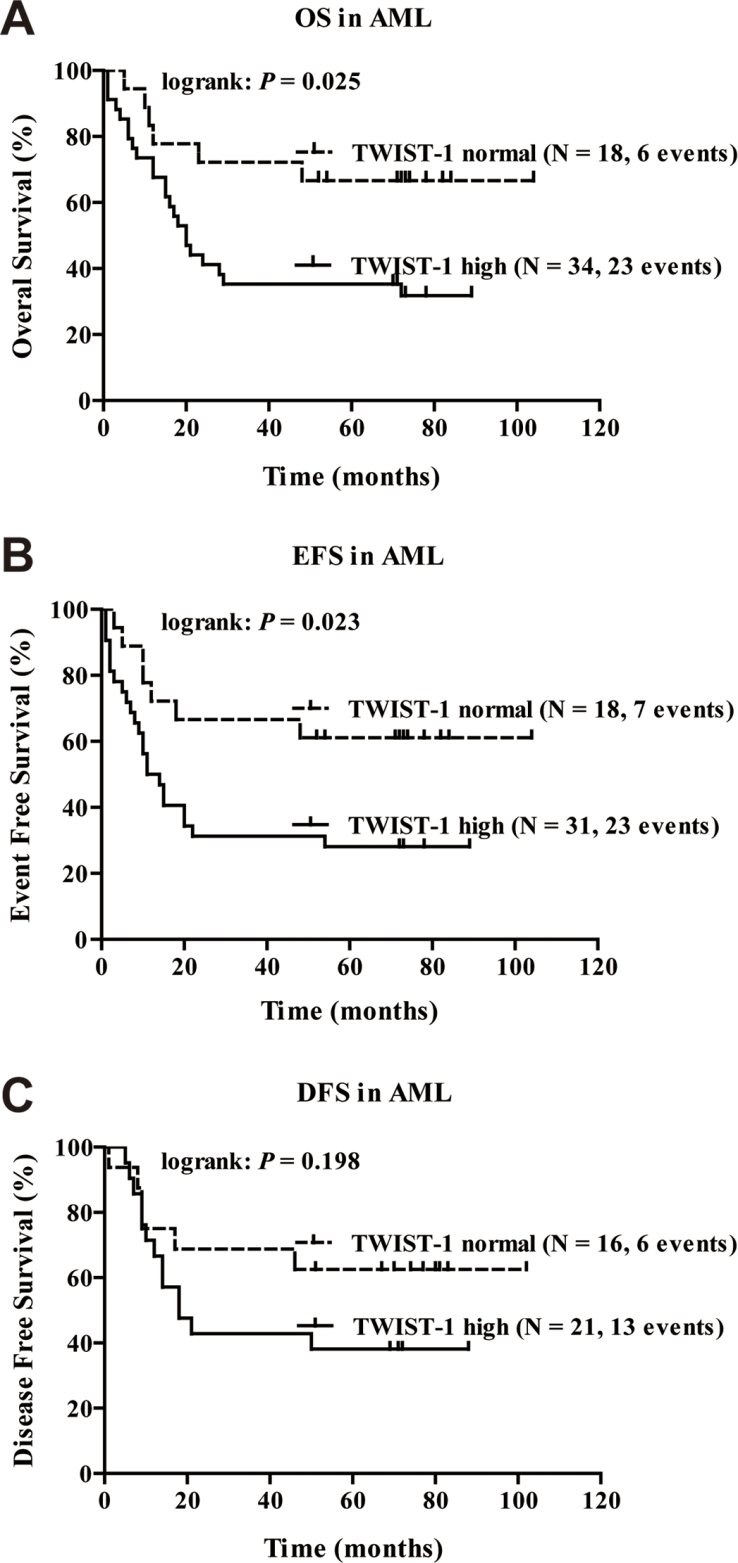
High TWIST-1 expression correlates with shorter OS and EFS in patients with AML Kaplan-Meier plots of OS **A.** EFS **B.** and DFS **C.** in patients with AML. *P* values were determined with the log-rank test. The number of patients included in the analyses is shown in brackets.

Next, we analyzed whether TWIST-1 expression level correlates with clinical outcome in myeloid leukemia. Due to the limitations of available material and feedback of CML patients, here we intensively focused on clinical implication and prognostic outcome of TWIST-1 in AML. We studied the relation between TWIST-1 expression at diagnosis and parameters of long-term outcome. Because the level of expression of TWIST-1 appears to be a continuum, we defined a threshold for high versus normal TWIST-1 expression based on the expression of TWIST-1 in normal BM (threshold: median expression in normal BM). When all the AML cases were analyzed together (regardless of the cytogenetic and phenotypic subgroup), we found that high TWIST-1 expression was associated with worse overall survival (OS), event-free survival (EFS) and disease-free survival (DFS). The 6-year OS was 66.7% ± 11.1% for patients with high TWIST-1 expression compared with 31.8% ± 8.1% for patients with normal TWIST-1 levels (*P* = .025, Figure 7A), the 6-year EFS was 61.1% ± 11.5% compared with 28.1% ± 7.9% (*P* = .023, Figure 7B) and the 6-year DFS was 62.5% ± 12.1% compared with 38.1% ± 10.6% (*P* = .198, Figure 7C), respectively.

To determine whether high TWIST-1 expression is an independent predictor for worse OS, EFS, and DFS, a multivariate analysis with the Cox proportional hazard model was performed. Starting with variables that were univariate predictors in this data set, a stepwise analysis was conducted until only significant variables remained. When entered into a multiple Cox regression analysis adjusting for these established prognostic factors, only TWIST-1 expression and age were independent prognostic factors for OS and EFS. We observed a significant correlation between high TWIST-1 expression with worse OS (HR = 2.893, *P* = .036, Table 1) and EFS (HR = 2.696, *P* = .037, Table 1). In conclusion, high TWIST-1 expression is a novel independent factor for adverse clinical outcome in AML.

**Table 1:** Multivariable analyses for overall survival and event-free survival in AML patients

Prognostic factor	Overall Survial			Event-free Survial		
	N	Univariate analysis: *P*	Multivariate analysis; HR (95% CI), *P*	N	Univariate analysis: *P*	Multivariate analysis; HR (95% CI), *P*
**TWIST-1 expression**						
High	33	.010	2.893 (1.07–7.80), .036	31	.018	2.696 (1.06–6.84), .037
Normal	17			17		
**Age**						
≥45	15	.042	2.638 (1.19–5.84), .017	15	.014	2.538 (1.16–5.57), .02
<45	33			31		
**Sex**						
Male	31	.125	-	31	.036	0.485 (0.20–1.16), .105
Female	19			18		
**Hemoglobin**						
≥90	21	.189	-	21	.285	-
<90	27			25		
**WBC**						
≥10	28	.006	1.995 (0.82–4.86), .129	27	.001	2.351 (0.91–6.06), .129
<10	20			19		
**Platelet**						
≥100	9	.156	-	9	0.116	-
<100	39			37		
**BM blasts (%)**						
≥90	4	.180	-	4	0.203	-
<90	30			29		
**PB blasts (%)**						
≥40	25	.231	-	23	0.145	-
<40	9			9		

## DISCUSSION

There is only a little information thus far on the expression and functional role of TWIST-1 in hematopoietic malignancies. Here we determined TWIST-1 expression in patients with AML, ALL and CML and provided the first evidence that TWIST-1 was highly expressed in patients with AML, whereas no significant difference was observed between ALL patients and controls. The finding that TWIST-1 is overexpressed in AML is further supported by the data from Oncomine expression database. We also demonstrated higher expression of TWIST-1 in patients with CML, which is consistent with the observation of Cosset et al [25]. In addition, gain-of-function and loss-of-function analyses revealed that high TWIST-1 expression promoted cell growth, colony formation and drug resistance in both AML and CML cell lines. Therefore, although myeloid leukemias are heterogeneous in terms of phenotype, disease progression, prognosis, and response to therapy, TWIST-1 appears to be a common pathogenic factor for both AML and CML, suggesting its importance in the development of myeloid leukemias.

LSCs are first identified in AML by John Dick and colleagues [38, 39] and are considered responsible for leukemia initiation, relapse and resistance to chemotherapy. Both AML and CML have been reported to be LSC diseases [3, 5]. Although some studies have reported that LSCs in some types of AML may be present within myeloid progenitor population [40, 41], the CD34^+^CD38^−^ population concentrates the LSCs in a vast majority of cases [42], and is thought to contain very primitive HSCs with long-term reconstitution activity and multipotent progenitor cells [43]. Here we observed that TWIST-1 was mainly expressed in immature CD34^+^CD38^−^ cells from patients with AML and CML but that expression declines with differentiation. TWIST-1 knockdown in AML and CML CD34^+^ cells inhibited their proliferative capacity, indicating that TWIST-1 might be a key factor in the maintenance of LSCs function. These findings also provide a possible mechanism by which TWIST-1 is involved in the development of both AML and CML. Our previous study has revealed that TWIST-1 is predominantly expressed in LT-HSC in normal adult mouse BM, and it is involved in the maintenance of HSC dormancy and self-renewal capacity. Here we found that TWIST-1 also had higher expression in immature human CB CD34^+^ cells than in CD34^−^ cells (Supplementary Figure S7A). Down-regulation of TWIST-1 resulted in a significant inhibition in the colony forming ability in CB CD34^+^ cells (Supplementary Figure S7B). These observations suggest that TWIST-1 may be involved in regulation of both LSCs and HSCs. Several factors spanning Notch, HOX genes and the polycomb proteins are not only associated with self-renewal of HSC, but also play a role in the perpetual self-renewal of LSC [44–49]. TWIST-1 tends to be a new member of these regulatory factors. Of note, we demonstrated that TWIST-1 expression level was significantly higher in CD34^+^CD38^−^ immature cells from AML and CML patients compared with those from CB, suggesting that although TWIST-1 plays roles in both leukemia and normal stem cells, it may be functionally more critical for leukemia than for normal stem cells [50].

We performed transcriptome analysis to obtain clues of how TWIST-1 affected myeloid leukemia. Through the array data, we focused on a tiny set of overrepresented genes. Real-time PCR analysis validated 5 candidates, namely c-MPL, TRIB3, NUMB, SMAD3 and JMJD1C, and c-MPL was found to be the most significantly upregulated in TWIST-transfected U937 cells. Myeloproliferative leukemia virus proto-oncogene c-MPL is a gene encoding the receptor for the cytokine thrombopoietin (TPO). It has been reported that c-MPL is highly expressed in LSCs of AML and high level of TPO/c-MPL signaling in AML enhances cell resistance to chemotherapy, survival and self-renewing capacity [32, 34, 35, 51–53]. In addition, c-MPL overexpression is an adverse prognostic factor associated with shorter complete remission duration and unfavorable cytogenetics in AML [33, 54, 55]. Here we demonstrated the positive correlations exist between TWIST-1 and c-MPL expression in AML and CML cells. Functional studies indicate that c-MPL is an important intermediary in TWIST-1-mediated leukemogenic effects. Intriguingly, although K562 and U937 were not grown in the presence of TPO, the increased phosphorylation PI3K/AKT and JAK2/ERK pathways upon TWIST-1 and c-MPL overexpression were observed, suggesting the activation of c-MPL. One possible explanation is that TPO is produced by leukemia cells before or after TWIST-1 and c-MPL transduction. We then measured TPO levels by sandwich ELISA in the culture supernatant of wild type, vector-, TWIST-1- and c-MPL-transfected K562 and U937 cell lines, and found that TPO levels were undetectable in these groups (data not shown). It has been reported that there are some factors could overlap the biological effects with TPO by interacting with c-MPL [56]. Additional studies are required to uncover the mechanism by which TWIST-1 activates c-MPL without TPO.

Efforts are being made in recent years to identify new prognostic markers in order to help in deciding the route of treatment for the patients. In the previous reports, expression of TWIST-1 is a statistically significant prognostic factor in several types of solid tumors, where high expression of TWIST-1 resulted in a worse patient outcome [10, 12, 14, 57, 58]. In our study, we evaluated the prognostic significance of TWIST-1 mRNA expression levels in 52 adult patients with AML. Our data revealed that TWIST-1 overexpression was associated with treatment failure, specifically shorter OS and EFS. To estimate the contribution of prognostic factors significance in univariate analysis, multivariate analysis was performed. It showed that TWIST-1 expression levels and age were both independent prognostic parameters for OS and DFS in patients with AML. For the first time, TWIST-1 was verified as an independent prognostic marker in AML. However, the limitation in this study is that we have not included cytogenetic and molecular abnormalities prognostic marker in our analysis because of the limited patient material. Hence, the clinical significance of TWIST-1 expression needs to be further evaluated in large clinical trials in the context of other prognostic markers.

In conclusion, our data substantiate the important pathogenic roles for TWIST-1 in myeloid leukemia and provide a new molecular marker for refining the risk classification of AML. In the future, TWIST-1-targeted therapy may represent a potential new approach for patients with AML and CML who have higher expression of this protein.

## MATERIALS AND METHODS

### Cell lines and reagents

See Supplementary Materials and Methods for details.

### Patient samples and cord blood cell isolation and culture

BM specimens were collected from patients with a new diagnosis of AML (*n* = 103), CML (*n* = 59), or ALL (*n* = 37) at Institute of Hematology and Blood Diseases Hospital, Chinese Academy of Medical Sciences from January 2005 to December 2008, which were under the ethical principles for medical research and approved by the Ethics Committee of Institute of Hematology and Blood Diseases Hospital. Informed consent was obtained in accordance with Declaration of Helsinki. BM cells from healthy volunteers and patients with ITP, a benign hematological disease, were used as controls (*n* = 29). CB was obtained after informed consent from healthy postpartum women. MNCs were isolated by Ficoll-Hypaque density gradient centrifugation. CD34 and CD38 cells were selected by immunomagnetic column separation (Miltenyi Biotech, Auburn, CA) following the manufacturer's instructions. Cells were cultured in IMDM Medium (Invitrogen, Carlsbad, CA) supplemented with 5% fetal bovine serum (Invitrogen), 100 ng/mL SCF, 50 ng/mL TPO, and 100 ng/mL Flt-3 (PeproTech, Rocky Hill, NJ) at 37°C in 5% CO_2_.

### RNA extraction and quantitative real-time PCR

Total RNA was isolated using the TRIzol extraction reagent (Invitrogen) or an RNeasy Mini Kit (Qiagen, Hilden, Germany). cDNA was synthesized with M-MLV Reverse Transcript reagent (Invitrogen). Quantitative real-time PCR was performed with SYBR Green PCR kit (TaKaRa Bio Inc, Otsu, Shiga, Japan) and analyzed in an ABI 7500 Sequence Detection System. The expression level of each gene was normalized to the expression level of GAPDH. PCR primers were listed in Supplementary Table S1.

### Immunohistochemical staining, Western blot analysis and FACS analysis for protein detection

See Supplementary Materials and Methods for details.

### Lentivirus and Retrovirus vector production and transduction

According to the human TWIST-1 mRNA sequence, TWIST-1 shRNA sequences were designed using online RNAi design software. The non-interference sequences were composed of the same bases as the TWIST-1 shRNA and served as controls. The sequences of the shRNA oligos were listed in Supplementary Table S1. The constructs pLL3.7-shTWIST-1a, pLL3.7-shTWIST-1b, pLL3.7-shcontrola and pLL3.7-shcontrolb were derived by single-strand annealing of the oligonucleotides of the target sequences and subsequent cloning of the double-stranded oligonucleotides into the pLL3.7 vector. pLL3.7-shcontrol and pLL3.7-shTWIST-1 were named as shCtrl and shTWIST-1 in our study, respectively. To create the pMSCV-IRES-BFP vector, the green fluorescent protein (GFP) in the pMSCV-IRES-GFP backbone was replaced with blue fluorescent protein (BFP). The cDNAs for c-MPL was cloned into pCDH1-MCS1-EF1-copGFP (System Biosciences, Mountain View, CA) and pMSCV-IRES-BFP transfer vectors, respectively. The resulting constructs were verified by sequencing. Vectors were transfected into 293T cells using Lipofectamine 2000 (Invitrogen). Supernatant containing retroviruses was collected and concentrated 48 and 72 hours later and was used for the transduction. The transduction of cells was done by spinning with viral supernatant. Seventy-two hours after transduction, cell sorting for GFP fluorescence was done by the FACS Aria II instrument (BD Biosciences, San Jose, CA).

### Cell proliferation, clonogenic assay, animal xenograft tumor model, cell cycle, apoptosis assay, cell viability assay and luciferase reporter assay

See Supplementary Materials and Methods for details.

### Colony-forming cell assay

For the CFC assay, purified CD34^+^ cells were transduced with shCtrl or shTWIST-1 virus, and then sorted. A thousand GFP^+^ sorted cells were suspended in methylcellulose media (MethoCult H4434; Stemcell Technologies, Vancouver, BC, Canada) and then incubated at 37°C for 14 days. A colony was defined as a cluster of more than 50 cells. For the CML samples, colonies were pooled, and the percentage of BCR/ABL-positive cells was determined by PCR. A genomic PCR for the detection of FLT3/ITD was performed on colonies plucked from direct CFC assays. PCR primers were listed in Supplementary Table S1.

### Array analysis

Control and TWIST-1 transduced U937 cells were harvested for array analysis using Affymetrix GeneChip Human Transcriptome Array 2.0. This dataset has been disposed to Gene Expression Omnibus (GEO), and the accession number is GSE68362. Data were normalized with quantile normalization; a differential expression of at least a 1.2-fold change was used to define up- and down-expression.

### Statistical analysis

Statistical analyses were performed with SPSS software, release 17.0. The association between variables was analyzed by the χ^2^-square and the Fisher's exact test for categorical variables and the Student's *t* test or the Mann-Whitney *U* test for continuous variables. Correlations between continuous variables were calculated using the Spearman correlation or Logistic correlation analyses. To assess outcome, the following parameters were used: OS (defined as the time from diagnosis to death of any cause or end of follow-up); EFS (defined as the time from diagnosis until first event, in which failure to achieve complete remission, relapse, death, or end of follow-up were considered events); DFS (defined as the time between achieving complete remission and relapse). OS, EFS, and DFS were estimated according to Kaplan-Meier and compared according to log-rank test. The association between TWIST-1 and OS, EFS and DFS was tested in univariate Cox models. Cox regression analysis was applied to determine the association of TWIST-1 expression and OS/EFS/DFS with adjustment for risk factors such as age, white blood cell count, sex. The parameters were considered either as continuous or as categorical variables and only those reaching a *P* value < 0.1 in the respective univariate analysis were included. All *P* values were considered significant when < 0.05.

## SUPPLEMENTARY INFORMATION


