# Supplementary material for: TWIST-1 promotes cell growth, drug resistance and progenitor clonogenic capacities in myeloid leukemia and is a novel poor prognostic factor in acute myeloid leukemia
Source: Oncotarget. 2015 May 20;6(25):20977–92. doi: 10.18632/oncotarget.4007 (PMC4673244; doi:10.18632/oncotarget.4007)
Supplement: Supplementary file 1 [file oncotarget-06-20977-s001.pdf]

SUPPLEMENTARY INFORMATION

SUPPLEMENTARY MATERIALS AND METHODS

Cell lines and reagents

The following human hematopoietic malignant cell lines were used in this study: K562 (chronic myeloid leukemia), KG1a (acute myelogenous leukemia), U937 (promonocytic leukemia), HL-60 and NB4 (promyelocytic leukemia), J6-1 (human monocytic leukemia), Jurkat (acute T lymphoblastic leukemia), CEM (T lymphoblastic leukemia), Namalwa and Ramos (B-cell lymphoma). These cell lines were obtained directly from the American Type Culture Collection (ATCC) (University Boulevard Manassas, Virginia, USA) and were passaged in our laboratory for less than 6 months after receipt. The ATCC characterized these cell lines by DNA profiling.

Imatinib, Daunorubicin (DNR) and Mitoxantrone (MXT) were purchased from Sigma-Aldrich (Louis, MO).

Immunohistochemical staining for TWIST-1 protein

TWIST-1 protein expression was determined by immunohistochemical (IHC) staining. BMMNCs were fixed using 4% paraformaldehyde solution. IHC was performed using mouse anti-human TWIST-1 monoclonal antibody (Abcam, Cambridge, UK) and followed by the avidin-biotin peroxidase procedure. Expression of TWIST-1 protein was semi-quantitatively assessed by IHC score. The IHC score was the product of staining intensity (no staining = 0, weak staining [light yellow] = 1, moderate staining [yellowish brown] = 2, strong staining [brown] = 3) and the percentage of positively stained cells.

Proliferation assay

Cell proliferation was assessed by a growth curve, which was generated by seeding cells at 1×10^4 cells/ml for K562 and 1×10^5 cells/ml for U937, KG1a or Jurkat and counting cells using a hemocytometer under light microscopy by trypan blue exclusion method at 24 hours intervals for 8 or 9 days.

Clonogenic assay

Transfected K562 (2×10^3 cells/ml), KG1a (5×10^3 cells/ml) or U937 cells (5×10^3 cells/ml) were seeded into 0.9% methylcellulose medium. Colonies were counted after 14 days using an inverted microscope. A colony was defined as a cluster of more than 50 cells.

Animal xenograft tumor model

Fourteen NOD/SCID mice were irradiated with 300 cGy and were randomly assigned to two groups. A suspension of 1×10^7 transfected K562 cells in 500 μl PBS was injected by tail vein. The survival time was observed and the fraction of human CD13 and CD33 (BD Biosciences) positive tumor cells in BM and spleen was analyzed by FACS. All animal experiments were approved by the institute's Animal Research Committee.

Cell cycle and apoptosis assay

For cell cycle analyses, cells were fixed in cold ethanol, stained with propidium iodide or Ki67 and analyzed by flow cytometry. Apoptosis analysis was performed with PE-labeled Annexin-V and 7-AAD (BD Biosciences) according to the manufacturer's instructions.

Western blot analysis

Western blot analysis was done by standard techniques. Proteins were detected with primary antibodies against TWIST-1 (Abcam), PI3K (Cell Signaling, Danvers, MA, USA), p-PI3K (Cell Signaling), ERK (Cell Signaling), p-ERK (Cell Signaling), STAT3 (Cell Signaling), p-STAT3 (Cell Signaling), STAT5 (Cell Signaling), p-STAT5 (Cell Signaling), or β-Actin (Santa Cruz Biotechnology, Santa Cruz, CA).

Cell viability assay

Cell viability was determined by measuring the mitochondrial conversion of 3-(4,5-dimethylthiazolyl-2)-2,5-diphenyltetrazo-lium bromide (MTT; Sigma-Aldrich) to a colored product. The cells under disposal and their corresponding controls were plated in 96-well plates at a density of 2×10^4/0.1 ml for K562 and 4×10^4/0.1 ml for U937 cells and further incubated for 24 hours. The medium was then removed and replaced with fresh medium containing Imatinib, DNR or MXT at various concentrations for 24, 48 and 72 hours. After that, cells were stained with 20 μl MTT at 37°C for 4 hours followed by removal of culture medium and mixing of 150 μl DMSO thoroughly for 10 minutes. Spectrometric absorbance at 570 nm was measured with a microplate reader. Each group contained 3 wells. The half maximal inhibitory concentration value was determined by the dose of drug that causes 50% cell viability.

FACS analysis for c-MPL expression

The antibody of anti-human c-MPL PE (Miltenyi Biotech) was used for analysis of c-MPL expression in GFP^+U937 and GFP^+K562 cells at 3 days after the beginning of lentivirus transduction. Flow cytometry was performed on the Aria II instrument. Data analysis was carried out using FlowJo (Tree Star, Inc., Ashland, OR) software.

Luciferase reporter assay

For luciferase reporter experiments, the 3′-UTR segments of c-MPL predicted to interact with TWIST-1 were amplified by PCR from human cDNA and inserted into the pGL3 vector immediately downstream from the stop codon of luciferase (Promega, Madison, WI, USA). 293T cells seeded in 6-well plate were cotransfected with pCDNA3.1-TWIST-1-GFP or pCDNA3.1-vector-GFP and 50 ng of pGL3-c-MPL-3′UTR by using Lipofectamine-2000 transfection reagent. Luciferase activity was measured by Dual-Luciferase Reporter Assay System after 48 hours.

SUPPLEMENTARY FIGURES AND TABLES

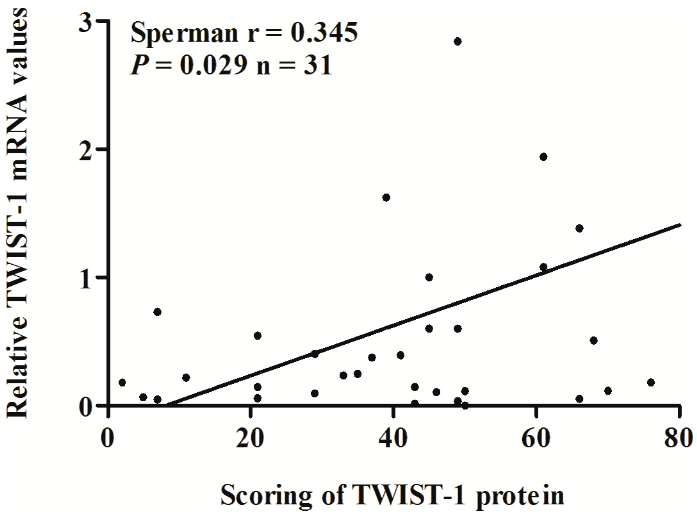

Supplementary Figure S1: Positive correlation between TWIST-1 mRNA expression and protein expression in patients with leukemia. The scatter plot of protein values versus mRNA values. The scoring of TWIST-1 protein in BMMNCs was measured by immunohistochemical staining and the value of TWIST-1 mRNA was quantified by quantitative real-time PCR. *P* value was determined with Spearman's rank correlation.

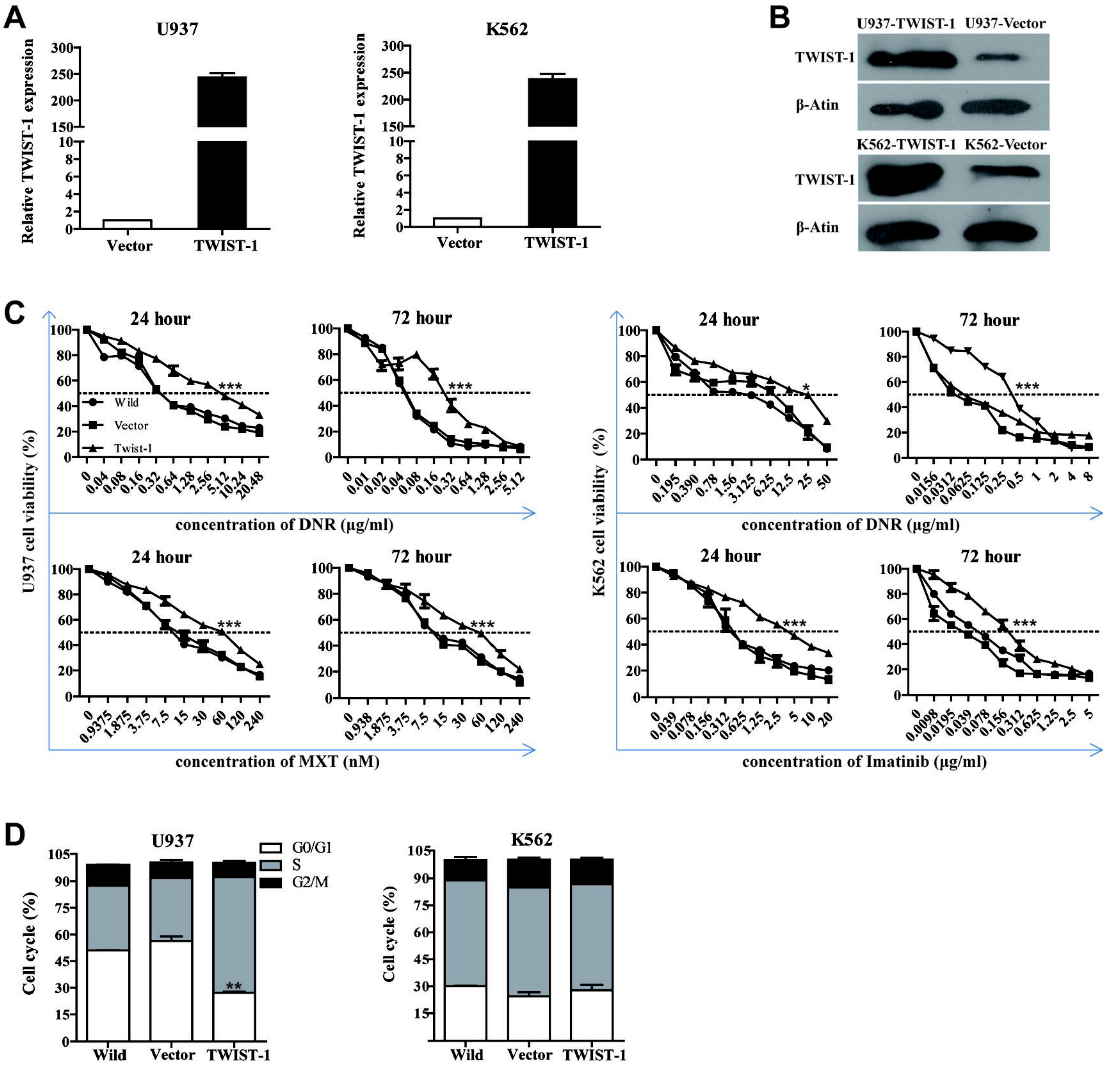

Supplementary Figure S2: TWIST-1 overexpression enhances the drug resistance and induces the accumulation of U937 cells in the S phase. A. Expression level of TWIST-1 transcripts in transduced GFP$^+$ cells using real-time PCR. **B.** Western blot analysis of TWIST-1 in the transduced GFP$^+$ cells. β-Actin is shown as equal loading. **C.** Transfected U937 and K562 cell lines were treated with various concentrations of DNR and MXT, or DNR and Imatinib respectively for 24 hours and 72 hours. Cell viability was measured by MTT assay and expressed as a percentage relative to control cells. IC50 values were calculated with XLfit software, which are marked in the middle of the box. **D.** Flow cytometry was used to compare the DNA content between control and TWIST-1-overexpressing cells. Summary of cell proportions in different phases of cell cycle. The results were expressed as mean ± SEM of three independent experiments. Asterisks denote significance (*, $P < 0.05$; **, $P < 0.01$; ***, $P < 0.001$).

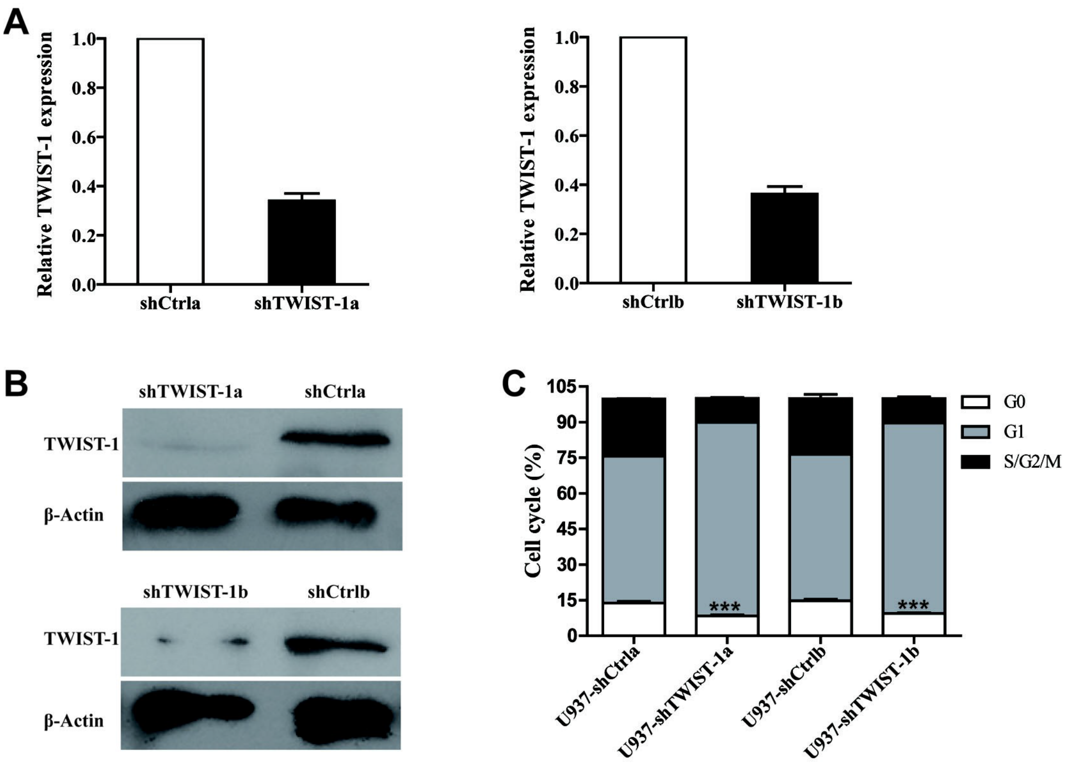

Supplementary Figure S3: TWIST-1 knockdown promotes cell cycle arrest in U937 cells. Cells were transduced with shCtrla, shTWIST-1a, shCtrlb or shTWIST-1b particles. GFP^{+} cells were sorted after 72 hours. **A.** Relative expression of TWIST-1 in K562 cells was analyzed by quantitative real-time PCR, as compared with control cells. Data are presented as mean ± SEM of three independent experiments. **B.** Western blot analysis of TWIST-1 in the transduced K562 cells. β-Actin is shown as equal loading. One representative blot is shown. **C.** Cell-cycle distribution of TWIST-1-silenced cells and non-silencing control cells was analyzed in U937 cells. The results were expressed as mean ± SEM of three independent experiments. Asterisks denote significance (***, $P < 0.001$).

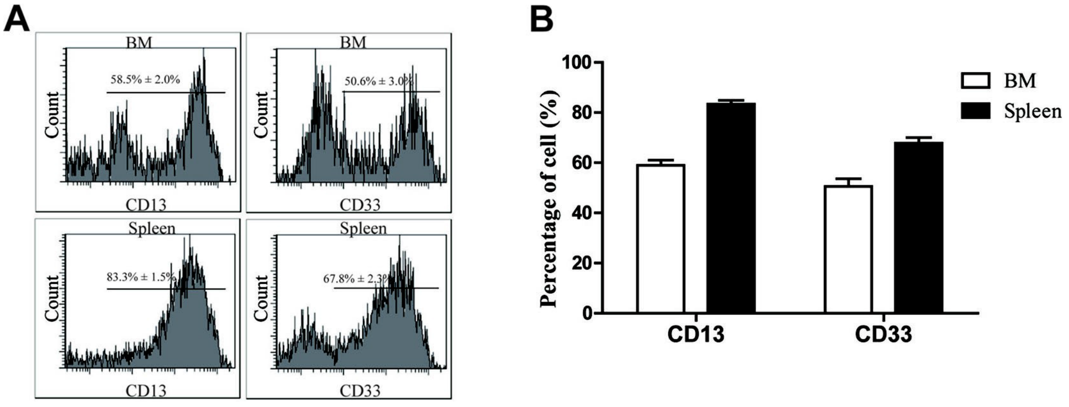

Supplementary Figure S4: FACS examines the expression of K562 marker in leukemic mice. A. Representative FACS plots showing CD13^{+} and CD33^{+} cells of BM and spleen in dying mice. **B.** Percentages of CD13^{+} and CD33^{+} cells of BM and spleen in dying mice (mean ± SEM, $n = 3$).

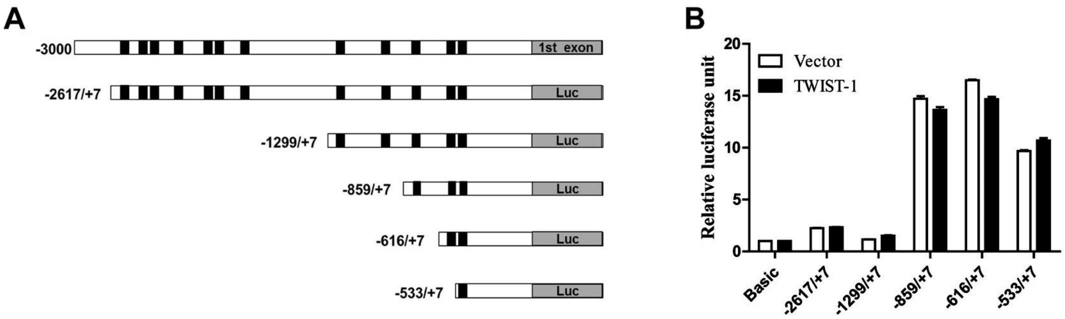

Supplementary Figure S5: c-MPL is not a direct target of TWIST-1. A. The 3′-UTR fragments of c-MPL were PCR-amplified and cloned. **B.** TWIST-1 overexpression did not affect the luciferase activity of c-MPL.

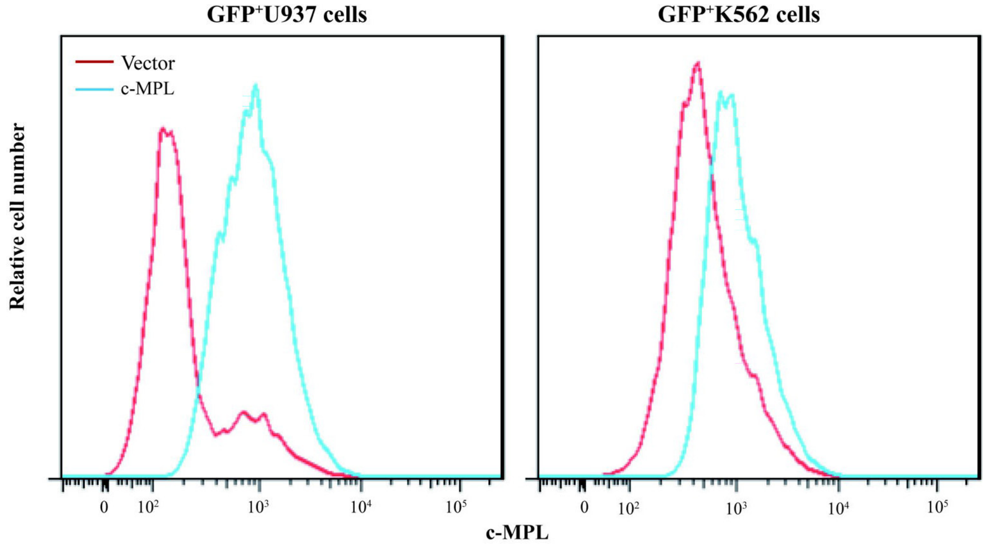

Supplementary Figure S6: FACS analysis of c-MPL in the transduced GFP^{+} cells.

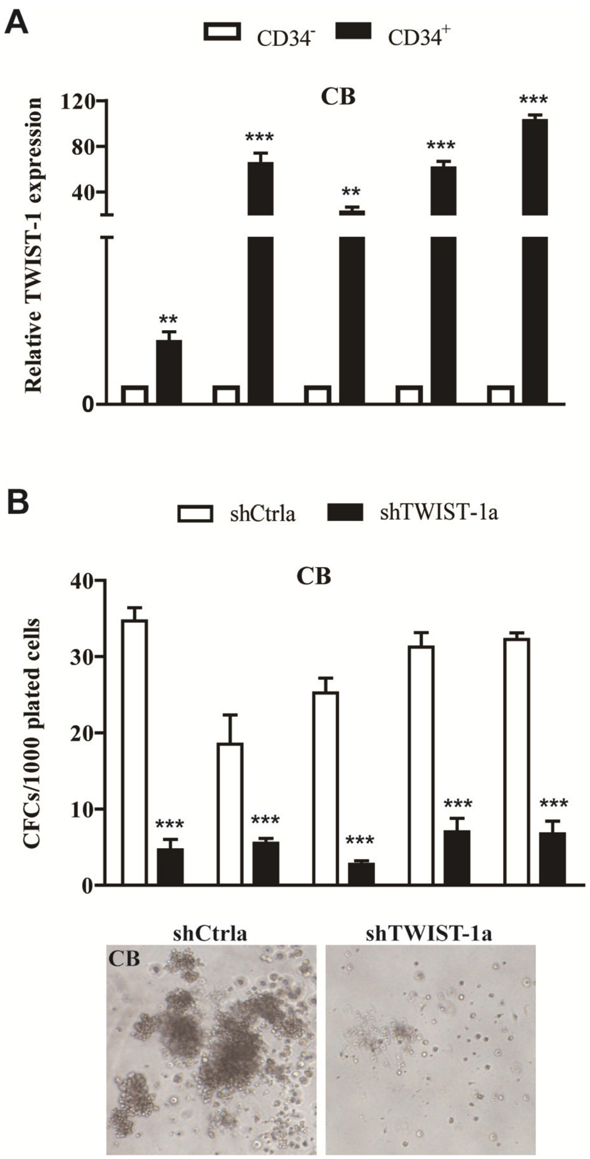

Supplementary Figure S7: TWIST-1 is highly expressed in CB CD34⁺ cells and knockdown of TWIST-1 impairs progenitor colony-forming capacity. A. Analysis of TWIST-1 expression in human CB $CD34^+$ and $CD34^-$ counterparts by quantitative real-time PCR. The TWIST-1 expression levels were normalized to that of GAPDH. Data are presented as mean ± SEM. **B.** CB $CD34^+$ cells were transduced with shCtrl or shTWIST-1 particles, sorted and plated 1,000 GFP-positive cells into methylcellulose medium H4434. After a14 days of culture, colonies were counted and morphologies of colonies were photographed. Data are presented as mean ± SEM. Asterisks denote significance (**, $P < 0.01$; ***, $P < 0.001$).

Supplementary Table S1: Oligonucleotide sequences

Name	Forward Primer (5′→3′)	Reverse Primer (5′→3′)
TWIST-1	GTCCGCAGTCTTACGAGGAG	TGGAGGACCTGGTAGAGGAA
GAPDH	GAAGGTGAAGGTCGGAGTC	GAAGATGGTGATGGGATTTC
c-MPL	TTTCTCCCGAACATTTGAGG	GTGCAGCGGAAAGAAGAGAC
TRIB3	TGCCCTACAGGCACTGAGTA	GTCCGAGTGAAAAAGGCGTA
AKT2	ACAGCAAAGCAGGAGTATAAG	AAAGAGCAGGAAACTACCAAT
CDKN2D	GTGCATCCCGACGCCCTCAAC	TGGCACCTTGCTTCAGCAGCTC
SMAD3	TGGACGCAGGTTCTCCAAAC	CCGGCTCGCAGTAGGTAAC
BCL-2	TGAACCGGCATCTGCACAC	CGTCTTCAGAGACAGCCAGGAG
RUNX1	CGAAGACATCGGCAGAAACT	TAAAGGCAGTGGAGTGGTTCA
BMI-1	GTATTCCCTCCACCTCTTCTTG	TGCTGATGACCCATTTACTGAT
YB-1	AAGTGATGGAGGGTGCTGAC	TTCTTCATTGCCGTCCTCTC
NUMB	CTTTTACAAGAGAAGGATCATT	CAACGACTATCTTATCTGTTTCAG
JMJD1C	CGACGCAGGTCTCGTGCCAA	TGGGCACGTGTATAATGGCTGTG
BCR/ABL1 p190	GACTGCAGCTCCAATGAGAAC	GTTTGGGCTTCACACCATTCC
BCR/ABL2 p190	CAGAACTCGCAACAGTCCTTC	TTCCCCATTGTGATTATAGCCTA
BCR/ABL1 p210/p230	GAAGTGTTTCAGAAGCTTCTCC	GTTTGGGCTTCACACCATTCC
BCR/ABL2 p210/p230	CAGATGCTGACCAACTCGTGT	TTCCCCATTGTGATTATAGCCTA
FLT3/ITD	GCAATTTAGGTATGAAAGCCAGC	CTTTCAGCATTTTGACGGCAACC
shTWIST-1a	GATCCGATGGCAAGCTGCAG CTATTTCAAGAGAATAGCTG CAGCTTGCCATCTTTTTG	AATTCAAAAAGATGGCAAGC TGCAGCTATTCTCTTGAAATA GCTGCAGCTTGCCATCG
shTWIST-1b	GATCCGCTGAGCAAGATTCA GACCTTCAAGAGAGGTCTGA ATCTTGCTCAGCTTTTTG	AATTCAAAAAGCTGAGCAAG ATTCAGACCTTCAAGAGAGG TCTGAATCTTGCTCAGCG
shCtrla	GATCCATGCGAGACGTACGC ATTGTTCAAGAGACAATGCG TACGTCTCGCATTTTTTC	AATTCAAAAAATGCGAGACG TACGCATTGTCTCTTGAACAA TGCGTACGTCTCGCATG
shCtrlb	GATCCATGCTACTAGACGCG AACGTTCAAGAGACGTTCGC GTCTAGTAGCATTTTTTG	AATTCAAAAAATGCTACTAG ACGCGAACGTTCAAGAGAC GTTCGCGTCTAGTAGCATG

Supplementary Table S2: Clinical characteristics of the 199 patients with leukemia

Characteristic	No. (%)
**Diagnosis**	
ALL	37 (18.6)
CML	59 (29.6)
AML	103 (51.8)
**Sex**	
Male	120 (60.3)
Female	79 (39.7)
**Age**	
Less than 50 years	150 (75.4)
More than 50 years	47 (23.6)
No data	2
**White blood cell count (cells × 10^9/L)**	
Less than 50	95 (47.7)
Between 50 and 100	14 (7.0)
More than 100	48 (24.1)
No data	42
**Tissue infiltration**	
negative	103 (51.8)
positive	60 (30.2)
No data	36
**Bone marrow blasts at diagnosis (%)**	
Less than 60	89 (44.7)
More than 60	68 (34.2)
No data	42

Supplementary Table S3: Comparison of clinical manifestations and laboratory features between patients with AML with lower and higher TWIST-1 expression

Variables	Total (*n* = 103)	Lower TWIST-1 expression (*n* = 51)	Higher TWIST-1 expression (*n* = 52)	*P*
**Sex, *n* (%)**				.079*
Male	62	30 (58.8)	32 (61.5)	
Female	41	21 (41.2)	20 (38.5)	
**Age, y**				.966
Mean	37	35	38	
Range	15–74	17–72	15–74	
**FAB subtype, *n* (%)**				
M1	1	1 (2)	0 (0)	‡
M2	23	11 (21.6)	12 (23.1)	.854*
M3	34	12 (23.5)	22 (42.3)	.043*
M4	7	2 (3.9)	5 (9.6)	.449*
M5	31	20 (39.2)	11 (21.2)	.046*
M6	3	3 (5.9)	0 (0)	‡
Missing	4	2 (3.9)	2 (3.8)	‡
**WBC count, × 10^9/L**				.183
Mean	14.59	17.27	12.39	
Range	0.69–267.2	0.79–267.26	0.69–193.1	
**% BM Blasts**				.917
Mean, %	56.75	56.75	56.25	
Missing, *n* (%)	17 (16.5)	9 (8.7)	8 (7.7)	
**CD34 expression, *n* (%)**				1.00*
CD34 low	68	34 (66.7)	34 (65.4)	
CD34 high	28	14 (27.5)	14 (26.9)	
Missing	7	3 (5.8)	4 (7.7)	

The median value of TWIST-1 expression in total population was used as the cutoff point to define lower- and higher-expression groups.
WBC indicates white blood cell.
P values not otherwise indicated were calculated with Wilcoxon (Mann-Whitney) test and are for the comparisons between lower TWIST-1 expression and higher TWIST-1 expression.
* *P* values were calculated with Fisher exact test for the comparisons between lower TWIST-1 expression and higher TWIST-1 expression.

Supplementary Table S4: Comparison of clinical manifestations and laboratory features between patients with CML with lower and higher TWIST-1 expression

Variables	Total (*n* = 59)	Lower TWIST-1 expression (*n* = 29)	Higher TWIST-1 expression (*n* = 30)	*P*
**Sex, *n* (%)**				.351*
Male	33	18 (62.1)	15 (50.0)	
Female	26	11 (37.9)	15 (50.0)	
**Age, y**				.839
Mean	40.5	41.5	39.5	
Range	14–78	16–66	14–78	
**WBC count, × 10^9/L**				.435
Mean	127.24	120.7	131.44	
Range	2.5–431.43	2.5–408.94	28.24–431.43	
**% BM Blasts**				.076
Mean, %	15.4	25	8.5	
Missing, *n* (%)	14 (23.7)	3 (10.3)	8 (7.7)	
**Tissue Infiltration**				.324*
Negative	18	12 (41.4)	6 (20.0)	
Positive	27	14 (48.3)	13 (43.3)	
Missing	14	3 (10.3)	11 (36.7)	

Supplementary Table S5: FLT3/ITD and BCR/ABL detection in colonies from CFC experiments

	AML (FLT3/ITD$^+$ colonies/total colonies) (%)		CML (BCR/ABL$^+$ colonies/total colonies) (%)	
**Patients**	**shCtrl**	**shTWIST-1**	**shCtrl**	**shTWIST-1**
1	25/30 (83.3%)	4/6 (66.7%)	12/15 (80.0%)	4/6 (66.7%)
2	22/25 (88.0%)	5/6 (83.3%)	7/10 (70.0%)	1/3 (33.3%)
3	8/11 (72.7%)	2/3 (66.7%)	13/21 (62.0%)	3/6 (50.0%)
4	6/7 (85.7%)	0/1 (0%)	27/33 (81.8%)	8/13 (61.5%)
5	19/22 (86.4%)	3/5 (60.0%)	41/53 (77.3%)	7/12 (58.3%)
6	15/21 (71.4%)	2/4 (50.0%)		
